# Determination and Characterization of (Novel) Circulating Strains of *Brucella* sp. Within the National Bovine Brucellosis Control Program in Ecuador

**DOI:** 10.3390/pathogens14020158

**Published:** 2025-02-06

**Authors:** Ana Dolores Garrido-Haro, Merci Falconí, Paola Moreno-Caballeros, María Elena-Rovalino, Hugo Rosero-Mayanquer, Michelle Yugcha-Díaz, David Fretin, Constance Wielick, Claude Saegerman, Jorge Ron-Román

**Affiliations:** 1 Agencia de Regulación y Control Fito y Zoosanitario (AGROCALIDAD), Quito 170184, Ecuador; ana.garrido@agrocalidad.gob.ec (A.D.G.-H.); mercy.falconi@agrocalidad.gob.ec (M.F.); paola.moreno@agrocalidad.gob.ec (P.M.-C.); maria.rovalino@agrocalidad.gob.ec (M.E.-R.); hugo.rosero@agrocalidad.gob.ec (H.R.-M.); 2 Research Unit of Epidemiology and Risk Analysis Applied to Veterinary Science (UREAR-ULg), Fundamental and Applied Research for Animals & Health (FARAH) Center, Faculty of Veterinary Medicine, University of Liege, 4000 Liège, Belgium; cwielick@uliege.be; 3 Grupo de Investigación en Sanidad Animal y Humana (GISAH), Carrera Ingeniería Agropecuaria, Departamento de Ciencias de la Vida y la Agricultura, Universidad de las Fuerzas Armadas (ESPE), Sangolquí 171103, Ecuador; emyugcha@espe.edu.ec (M.Y.-D.); jwron@espe.edu.ec (J.R.-R.); 4 Department of Animal Infectious Diseases, Laboratory of Veterinary Bacteriology, National Institute for Public Health (Sciensano), 1050 Brussels, Belgium; david.fretin@sciensano.be

**Keywords:** *Brucella* spp., cattle, Ecuador, bacteriology, MLVA-16, PCR

## Abstract

Brucellosis is a zoonotic disease caused by bacteria of the *Brucella* species. This infectious disease represents a significant public health and economic challenge in many regions of the world, including Ecuador. *Brucella abortus* is the most common species in cattle. Transmission mainly occurs through direct contact with secretions, aborted fetuses, or contaminated reproductive fluids. In this study, to evaluate the circulating strains of *Brucella* in continental Ecuador, *Brucella* strains were cultured and isolated from retromammary lymph nodes and milk samples collected over the past three years from six Ecuadorian provinces within the National Brucellosis Program of Ecuador. *Brucella* cultures were performed on two specific media, CITA and Farrell, followed by molecular identification using PCR and multiple-locus variable-number tandem repeat analysis 16 (MLVA-16) diagnostic techniques. Out of a total of 25 retromammary lymph nodes collected at slaughterhouses and 50 milk samples obtained from serologically positive animals on farms, *Brucella* was isolated from 35 milk samples and 19 retromammary lymph node samples and identified as *Brucella abortus* by PCR. Subsequent MLVA-16 genotyping enabled accurate discrimination among the *Brucella* strains present in Ecuador. This study confirmed the presence of *Brucella abortus* strains of biovars 1 and 4 and, for the first time, detected the presence of biovar 2 in Ecuador. The isolation and accurate detection of *Brucella*, along with the implementation of advanced genotyping techniques, such as MLVA, are crucial for future epidemiological studies, outbreak tracing, and the development of control strategies to mitigate animal and human infection in Ecuador.

## 1. Introduction

Brucellosis is a disease of significant economic importance in production animals worldwide [1] and a reportable disease under the World Animal Health Information System [2]. It is caused by species of the Gram-negative bacterium *Brucella*, members of the genus *Brucella*, which is a facultative intracellular pathogen [3]. Globally, brucellosis remains a major cause of disease in humans and livestock. The prevalence of brucellosis in animal reservoirs directly influences its incidence in humans [3]. The highest incidence rates are found in the Middle East, the Mediterranean region, sub-Saharan Africa, China, India, Peru, and Mexico, with a significant increase in cases in Central and Southeast Asia. In contrast, countries in Western and Northern Europe, Canada, Japan, Australia, and New Zealand are considered free of the infectious agent [2].

According to the list of prokaryotic names withstanding in nomenclature (https://lpsn.dsmz.de/genus/brucella, accessed on 22 November 2024), numerous species have been described within the genus *Brucella*, including *Ochrobactrum* spp. in this database. However, merging *Ochrobactrum* spp. in the genus *Brucella* induced controversy with experts in brucellosis due to several differences between these two groups (i.e., structure, physiology, population structure, core-pangenome assemblies, genome structure, genomic traits, clinical features, treatment, prevention, diagnosis, genus description rules, and pathogenicity) [4]. Classical species of *Brucella* include *B. abortus*, *B. melitensis*, *B. suis*, *B. canis*, *B. ovis*, and *B. neotomae*, while newly identified species include *B. ceti*, *B. pinnipedialis*, *B. microti*, *B. inopinata*, *B. papionis*, and *B. vulpis* [4]. *Brucella abortus* is primarily predominant in cattle, *B. suis* in pigs, and *B. canis* in dogs. These species are traditionally divided into the following biovars: *B. melitensis* includes three biovars (1, 2, and 3); *B. abortus* contains eight (1, 2, 3, 4, 5, 6, 7, and 9); and *B. suis* contains five [5,6].

The *Brucella* genus is notably homogeneous, exhibiting over 90% genetic homology between different species within the same genus [7]. It is classified based on various phenotypic, genotypic, and ecological characteristics. Traditionally, *Brucella* species are identified by their primarily predominantly host and specific biochemical and serological traits [8].

The *Brucella* genus consists of coccobacilli, or short bacilli, measuring 0.6 to 1.5 μm in length and 0.5 to 0.7 μm in width. They are usually observed as isolated cells; although, they occasionally appear in pairs or small groups. While their morphology is fairly uniform, pleomorphic forms may occur in older cultures. *Brucella* is a non-motile bacterium, does not sporulate, and lacks true fimbriae or capsules [2]. However, recent research on *Brucella* has revealed the presence of genes related to flagellar formation in its genome. The expression and assembly of functional flagella are highly regulated and do not manifest under standard conditions, suggesting a potential latent motility or conditioned by specific environments; the expression of these flagellar genes is tightly controlled by environmental factors and transcriptional regulators that ensure that flagellar production only occurs under specific conditions [9]. Bacteriological culture is considered the gold standard for brucellosis diagnosis due to its high specificity and ability to identify different species and biovars. This method is crucial for epidemiological studies, as it helps track the spread of the disease within a region and identifies potential sources of infection [9,10]. However, it is a laborious process that requires specific biosafety measures due to the risk of infection for laboratory personnel [11]. Serologic assays are, therefore, useful tools for the diagnosis of brucellosis, especially in resource-limited settings. These assays allow for the rapid and relatively low-cost detection of antibodies directed against *Brucella*, though they may present issues related to sensitivity and specificity [12].

The detection of *Brucella abortus* by polymerase chain reaction (PCR) has established itself as an effective technique due to its high sensitivity and specificity. The IS711 insertion sequence, which is specific to the genus *Brucella*, allows the species of this genus to be identified and differentiated with great precision, making it a useful tool for diagnosis and genetic characterization [5]. First detailed by López-Goñi et al. in 2008, the Bruce-ladder is a multiplex PCR technique developed for the identification and differentiation of *Brucella* species, including those used for vaccines [13]. The multiple-locus variable-number tandem repeat analysis (MLVA) allows for discrimination between strains of *Brucella*, which is crucial for epidemiological studies, tracking outbreaks, and developing control strategies [7]. This technique amplifies specific *Brucella* DNA regions containing tandem repeats, followed by determining the number of repeats present [10]. In Latin America, brucellosis is a major zoonotic disease, posing significant public health and economic concerns. The disease is endemic in several countries across the region, affecting both animals and humans. Factors contributing to its prevalence include insufficient control and eradication measures in livestock, the consumption of unpasteurized dairy products, direct contact with infected animals, and in some regions, limited access to veterinary services [14]. The unregulated movement of animals or mixing of herds is a well-documented contributor to the spread of brucellosis, particularly in regions with limited biosecurity measures [3]. In addition, mixing production groups or mixing animals of different ages may facilitate disease transmission, as young or newly introduced animals may act as carriers of *Brucella* spp. and amplify spread within herds [2]. In addition, extensive beef herds raised on pastures, where different age and production groups intermingle, raise concerns regarding hygiene practices and disease prevention measures [14].

The Ecuadorian agricultural sector plays an important role in ensuring food sovereignty and contributes significantly to the national economy [15]. A national study conducted by the Agencia de Regulación y Control Fito y Zoosanitario—(AGROCALIDAD) reported an apparent prevalence of bovine brucellosis of 21.3% (95% CI: 16.8–26.6) at the herd level and 6.2% (95% CI: 5.5–7) at the animal level [16]. Risk factors identified through multivariate analysis included a herd larger than 70 hectares (odds ratio (OR) = 2.73, 95% CI: 1.18–6.32) and the number of calves per animal (two or more, with OR ≥ 1.8 and *p*-values ≤ 0.047) [16]. In the northwest of the country, the human seroprevalence of brucellosis was estimated at 1.88% (95% CI: 1.48–2.38), with the circulating strains identified as *Brucella abortus* biovar 4 [17] and *Brucella abortus* biovar 1 [18]. Controlling brucellosis in Ecuador remains a considerable challenge, requiring better coordination between the public health and veterinary sectors. The absence of a mandatory vaccination program, low biosecurity measures, inadequate farm infrastructure, limited resources, in addition to unregulated movement and inadequate testing are other important factors in the epidemiology of brucellosis in Ecuador, and low awareness in rural areas further hinders the effective control of the disease.

This study aims to implement essential methodologies in the national reference laboratory, including the culture and isolation of *Brucella* and the molecular typing of strains through MLVA. To achieve this, samples collected and stored between 2022 and 2024 were used. This approach allows for tracing the geographical distribution and spread of outbreaks, thereby enhancing epidemiological traceability in Ecuador.

## 2. Materials and Methods

### 2.1. Sampling Protocol

**Figure 1:**
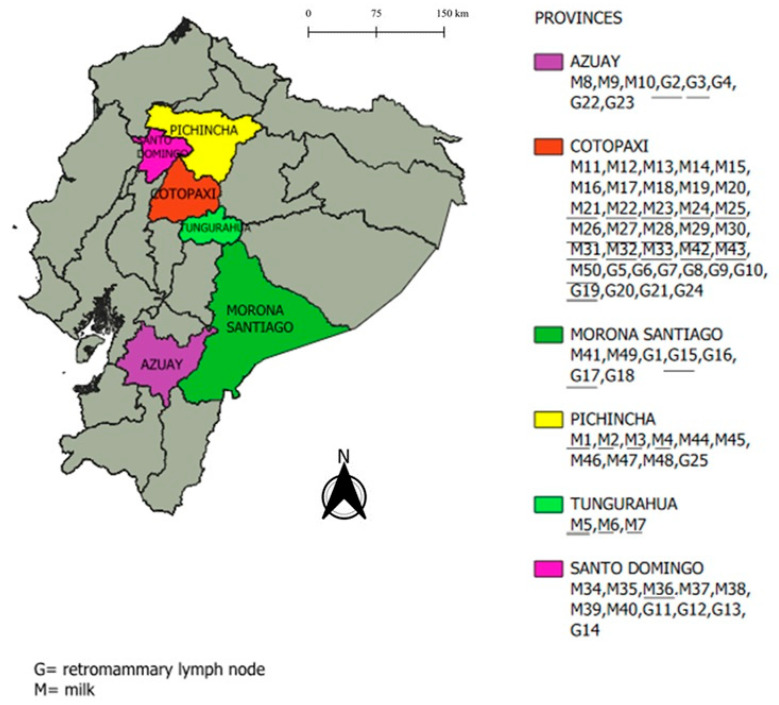
Map of continental Ecuador showing the six sampled provinces along with milk (M) and retromammary lymph node (G) samples collected from cattle in each province. Legend: Samples that are underlined correspond to those from which a strain of *Brucella abortus* was isolated with an MLVA-16 profile.

To implement the isolation and culture technique at AGROCALIDAD, 50 milk samples and 25 retromammary lymph node samples were collected from Rose Bengal and cELISA seropositive animals and stored between 2022 and 2024. The samples were obtained from the following six provinces: Morona Santiago, Azuay, Cotopaxi, Santo Domingo, Pichincha, and Tungurahua (Figure 1 and Table A1).

Two phases of sampling milk and retromammary lymph nodes were conducted on the farm (milk) and at slaughterhouses (lymph nodes). Milk was collected from lactating females in sterile containers, with approximately 25 mL taken from each quarter (100 mL in total). The lymph nodes were entirely and aseptically collected (without dissection) using sterile instruments and containers. In both cases, the samples were transported to the lab, while maintaining the cold chain.

### 2.2. Bacteriological Culture

#### 2.2.1. Sample Preparation

The samples were prepared for isolation in the containment laboratory BSL2+ at AGROCALIDAD, following the biosafety measures appropriate for the pathogen of interest.

Upon arrival at the laboratory, milk samples were refrigerated until phase separation became evident; subsequently, the supernatant was seeded. The tissue samples were surface disinfected by immersion in 95% ethanol, followed by a brief flaming. After disinfection, the fat was removed, and the tissue was cut into small pieces. The fragments were placed in sterile Stomacher bags, carefully avoiding damage to the bags. The samples were then homogenized for 1 min in a small amount of phosphate-buffered saline (PBS) to prevent excessive dilution, which could reduce the sensitivity of the culture. The homogenization process was conducted inside a biosafety cabinet.

Samples were inoculated in duplicate on Farrell selective medium and CITA medium (Centro de Investigación y Tecnología Agroalimentaria, Zaragoza, Spain), following the guidelines of [2,19]. Although Farrell medium effectively inhibits most contaminating microorganisms [20], it also suppresses the growth of *B. ovis* and some strains of *B. melitensis* and *B. abortus.* In contrast, CITA medium allows for the isolation of major *Brucella* species, including *B. suis* [2,19]. Volumes of 0.1–1 mL of the homogenized lymph node samples and 400 µL of milk sample supernatant were plated onto both Farrell and CITA media to enhance sensitivity, using a sterile swab or bacteriological loop, and streaked for isolation. The plates were incubated under appropriate conditions for 15 days at 37 °C ± 1 °C, with high humidity (near saturation) and 5–10% CO_2_.

#### 2.2.2. Morphological and Biochemical Characterization

On the third day, cultures were examined, and colonies suspected to be *Brucella* were observed under a stereomicroscope to assess their typical morphological characteristics. Following this evaluation, biochemical tests for oxidase, catalase, and urease were performed according to the protocols described by Alton et al. [21]. In cases where *Brucella* was confirmed, but a pure culture could not be obtained, the colonies were subcultured to non-selective media, such as tryptic soy agar (TSA), and incubated for at least 48 h to promote growth.

### 2.3. DNA Extraction

The isolated *Brucella* strains from positive cultures were inactivated by carefully transferring approximately six colonies into 500 μL of molecular biology-grade water, dissolving them, and heating the solution in a thermal block at 100 °C for 20 min. The solution was then centrifuged for 10 min at 8000 rpm, and the supernatant was collected. DNA extraction from the *Brucella* colonies was performed using the DNA extraction kit PureLink^TM^ Genomic DNA Mini Kit (Invitrogen^TM^, ref. K 182002, Waltham, MA, USA). The extracted DNA was quantified with a Thermo Scientific™ NanoDrop™ 2000 spectrophotometer (Waltham, MA, USA) and stored at −70 °C for further analysis.

### 2.4. PCR Assays

For the identification of *Brucella abortus* via PCR, the following primers were used: primer forward IS711 (5′-TGC CGA TCA CTT AAG GGC CTT CAT TGC CAG-3′) and primer reverse *Abortus* (5′-GAC GAA CGG AAT TTT TCC AAT CCC-3′) [5]. The final concentration of the components of the PCR reaction was as follows: Promega Go Taq Green Master Mix at 1×, at a concentration of 0.2 μM, and 5 µL of extracted DNA, resulting in a final volume of 50 µL. The PCR parameters consisted of an initial step at 95 °C for 3 min, followed by 34 cycles of 1 min and 15 s at 95 °C, 2 min at 55.5 °C, 2 min at 72 °C, and a final elongation step of 5 min at 72 °C.

Bruce-ladder PCR allows for the identification of classical *Brucella* species (*B. melitensis*, *B. abortus*, *B. suis*, *B. canis*, *B. ovis*, and *B. neotomae*) as well as vaccine strains in the sample (RB51, S19, and REV1) [13]. The components of the PCR reaction included 3 mM of MgCl_2_, 0.3 μM of primers 998F, 998R, 752F, 752 R, 987F, and 987R, and 0.12 μM of primers 535 F, 536R, 843F, 844R, 436F, 435R, 428F, 428R, 955F, and 953R, along with 1X Go Taq Master Mix (Promega, Madison, WI, USA) and 2 µL of extracted DNA. Primers are detailed in Table A2. PCR amplification was performed in a final volume of 25 μL. The temperature profile included an initial denaturation of 7 min at 95 °C, followed by 25 cycles of 95 °C for 35 sec, 64 °C for 45 sec, and 72 °C for 3 min, with a final elongation step of 6 min at 72 °C.

The reference materials used were DNA of strain 11,193 for the singleplex PCR and DNA of *Brucella suis* for the Bruce-ladder assay. Both were obtained from the Reference Laboratory for Brucellosis SENSA (Servicio Nacional de Sanidad y Calidad Agroalimentaria, Buenos Aires, Argentina). PCR products were analyzed on a 1.5% agarose gel by electrophoresis.

Finally, for the MLVA genotyping that enables molecular epidemiology studies, PCR was performed in a 20 μL reaction volume, with the final concentrations of the components comprising 1X Go Taq Master Mix (Promega), 2 μL of extracted DNA, and 0.3 μM of each primer from the MLVA-16 kit [7,22]. The thermal cycling conditions were as follows: an initial denaturation at 95 °C for 5 min, followed by 30 cycles of 96 °C for 30 s, 60 °C for 30 s, and 70 °C for 1 min, with a final elongation step at 70 °C for 10 min. Genotyping was carried out by analyzing minisatellite and microsatellite repeats, based on the MLVA-16 scheme. The tandem-repeat loci were divided into three groups: panel 1 (eight minisatellite loci: bruce06, bruce08, bruce11, bruce12, bruce42, bruce43, bruce45, and bruce55), panel 2A (three microsatellite loci: bruce18, bruce19, and bruce21), and panel 2B (five microsatellite loci: bruce04, bruce07, bruce09, bruce16, and bruce30) [6,7,22]. A 100 bp molecular weight marker (Invitrogen and Promega) was used for electrophoresis, and the size of each PCR product was converted to a corresponding tandem repeat number, as described by [7]. *Brucella* strain 2308 DNA served as the reference strain. The genotypes were compared with those in the MLVA bacterial genotyping database (https://microbesgenotyping.i2bc.paris-saclay.fr/databases/view/61/ (*Brucella* V4_6_5), accessed on 20 July 2024).

### 2.5. Statistical Analysis

To illustrate diversity within clusters based on the single-locus variation (SLV) [6], the MLVA profiles of the isolates were analyszed using a minimum spanning tree (MST) in BioNumerics version 6.6 software, where units, rather than sizes, of each marker were considered. Additionally, an MLVA-16 dendrogram of *Brucella abortus* was generated by geographic location in Latin America and biovars using R software version 4.4.1. The following libraries were used to generate the dendrogram: ape, phangorn, ggtree, readxl, and ggplot2.

## 3. Results

### 3.1. Morphological and Biochemical Characterization

The results of *Brucella* cultures on Farrell and CITA selective media showed that, out of 25 retromammary lymph node samples inoculated, 11 grew on Farrell medium, while 15 grew on CITA medium. Similarly, for the milk samples, 13 grew on Farrell medium; whereas, 33 grew on CITA medium (Table 1, Table A1 and Table A3). Overall, *Brucella* culture positivity was twice as high on CITA medium (N = 48) compared to Farrell medium (N = 24). Notably, for 6 samples (including 4 retromammary lymph nodes), growth was observed on Farrell medium but not on CITA medium; whereas, 30 samples were positive on CITA medium but not on Farrell medium.

The *Brucella* colonies observed under a stereomicroscope with backlighting exhibited a morphology characterized by a diameter of 1–2 mm, a translucent appearance, and a pale honey color. The colonies had well-defined edges and were slightly convex [2].

Biochemical tests showed that all isolates were positive for oxidase and catalase, with intermediate urease activity (the reaction occurring approximately after 3 h of incubation).

### 3.2. PCR Identification

**Figure 2:**
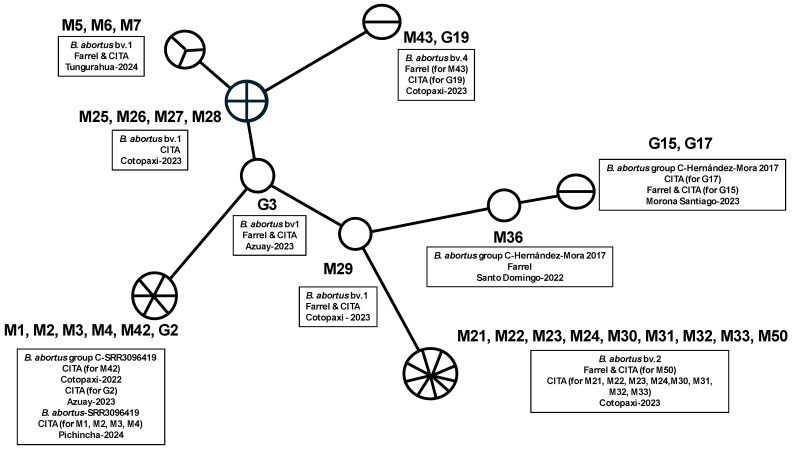
Minimum spanning tree generated from MLVA-16 genotyping of *Brucella* isolates from Ecuadorian cattle. Legend: Each strain is represented by a circle. Large circles group strains with identical genotypes, with the size of the circle proportional to the number of entries. The length of the lines connecting the different genotypes represent the number of mutations (VNTR—variable number tandem repeat).

**Figure 3:**
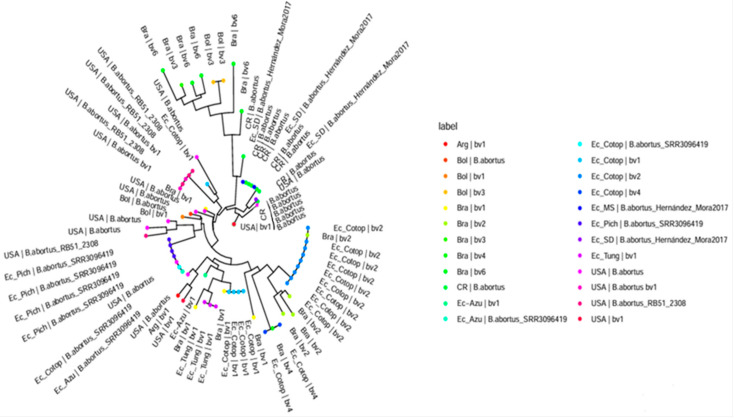
Dendrogram comparing the *Brucella abortus* strains identified in Ecuador with strains reported in different geographic locations in the Americas and their corresponding biovar. Legend: The analysis was performed using strains reported in the MLVA-16 database (https://microbesgenotyping.i2bc.paris-saclay.fr/databases/view/61 (accessed on 20 July 2024). Abbreviations: Arg, Argentina; Bol, Bolivia; Bra, Brazil; CR, Costa Rica; Ec_Azu, Azuay province of Ecuador; Ec_Cotop, Cotopaxi province of Ecuador; EC_MS, Morona Santiago province of Ecuador; Ec_Pich, Pichincha province of Ecuador; Ec_SD, Santo Domingo province of Ecuador; Ec_Tung, Tungurahua province of Ecuador; USA, United States of America; bv1, *B. abortus*; bv2, *B. abortus*; bv3, *B. abortus*; bv4, *B. abortus*; bv6, *B. abortus*; *B. abortus*_SRR3096419, reference strain from USA; *B. abortus*_Hernández_Mora2017 [26], reference strain from Costa Rica.

*Brucella abortus* was identified in milk and retromammary lymph node samples using Farrell and CITA media, as well as molecular techniques including *Brucella abortus*-specific PCR and Bruce-ladder multiplex PCR. Of the 50 milk samples tested, 35 were positive for *Brucella abortus*, with 13 isolates recovered on Farrell medium and 33 on CITA medium (Table A1). For retromammary lymph nodes, 19 of 25 samples were positive for *Brucella abortus*, with 11 isolates identified on Farrell medium and 15 on CITA medium (Table A3). Table 2 and Table A4 shows the results of the MLVA-16 assay on bovine isolates from Ecuador, revealing distinct *Brucella* biovars within the country. Figure 2 presents the minimum spanning tree generated from the MLVA-16 genotyping, while Figure 3 shows a dendrogram comparing the *B. abortus* strains identified in Ecuador with those reported in the MLVA-16 database from different geographic locations in the Americas and their corresponding biovar. Samples from Pichincha (M1, M2, M3, M4; 1 herd), Cotopaxi (M42; 1 herd), and Azuay (G2; 1 herd) were genotypically related, corresponding to *B. abortus*-SRR3096419 (https://www.ncbi.nlm.nih.gov/sra/SRR3096419, accessed on 18 August 2024) [23], and closely matched samples reported in the United States, themselves close to *B. abortus* biovar 1. Multiple isolates from Cotopaxi (M21, M22, M23, M24, M30, M31, M32, M50) across three different herds were also genotypically related and corresponded to B. ab+ortus biovar 2, showing similarities with genotypes found in Brazil [9,24,25]. Samples from Tungurahua (M5, M6, M7; 1 herd), Cotopaxi (M25, M26, M27, M28, M29; from two herds), and Azuay (G3; same herd as G2) were closely related to *B. abortus* biovar 1, clustering into four distinct genotypes closely related to *B. abortus* biovar 1 isolates from Brazil, Argentina, and the United States [9,24,25]. The M43 and G19 samples from Cotopaxi (2 herds) shared a similar genotype corresponding to *B. abortus* biovar 4, similar to one isolate found in Brazil (https://microbesgenotyping.i2bc.paris-saclay.fr/databases/view/61/, accessed on 20 July 2024). Finally, the samples from Santo Domingo (M36; one herd) and Morona Santiago (G15 and G17; two herds) were identified on the tree as two closely related genotypes, similar to *B. abortus* biovar group C-Hernández-Mora 2017 from Costa Rica [26].

## 4. Discussion

*Brucella abortus* is the causative agent of bovine brucellosis, a disease of major concern for both animal and public health, with significant economic impacts globally. In this study, lymph node and milk samples were collected from six provinces in Ecuador to isolate and genetically characterize *Brucella* strains. Using culture and PCR techniques, *Brucella abortus* was identified, and MLVA-16 analysis enabled differentiation into three biovars (1, 2, and 4). This research represents the first documentation of *B. abortus* biovar 2 in Ecuador, a finding that holds added value for epidemiological understanding and guiding future studies.

The use of two selective isolation media, such as Farrell’s and CITA’s media, is crucial for the microbiological diagnosis of *Brucella* bacteria, isolating *B. abortus* from bovine samples, allowing a higher recovery [2,21,27]. In Farrell’s medium [28], the concentrations of nalidixic acid and bacitracin can inhibit certain strains of *B. abortus*, *B. melitensis*, *B. suis*, *B. ovis*, and some other strains, including the vaccine strain RB51. Therefore, it is advisable to also use CITA medium, which contains vancomycin, colistin, nystatin, nitrofurantoin, and amphotericin B. CITA medium shows greater sensitivity to a wider range of *Brucella* species that may be present in the analyzed samples [2,19,29,30].

The study demonstrated that the CITA culture medium was more sensitive than Farrell’s medium for isolating *Brucella* from bovine samples, allowing for a higher recovery of the bacteria. Specifically, CITA medium supported positive growth in 48 cases, compared to 24 cases on Farrell’s medium, doubling the recovery rate. To optimize *Brucella* detection in surveillance studies, it is recommended to use both media in parallel whenever possible.

*Brucella abortus* biovar 1 is the most widely isolated strain in cattle worldwide [31]. In Latin America, it is the most frequently reported biovar, with documented cases in countries such as Argentina, Brazil, and Colombia. In Argentina, biovar 1 has historically been predominant and is associated with the high prevalence of bovine brucellosis, which is a significant animal and public health concern [32,33]. Similarly, *B. abortus* biovar 4 is common in Brazil, Colombia, Chile, Cuba, México, and Venezuela [9,25,33,34]. In Ecuador, *B. abortus* biovars 1 and 4 have been identified in previous studies [17,18]. In the present study, using the multi-locus VNTR analysis (MLVA), biovar 2 was identified for the first time in milk samples collected from Cotopaxi province. This biovar has been linked to natural infections in cattle, associated with “abortion storms” in infected gestating animals, neonatal death within hours, and stillbirths [33]. The confirmation of *B. abortus* biovar 2 presence in Ecuadorian cattle highlights the need for its surveillance within the national bovine brucellosis control and eradication program. In neighboring countries, like Bolivia and Peru, data on brucellosis are less well-documented [35]. National and regional collaborations are essential to achieve a more comprehensive understanding of brucellosis epidemiology.

Nine genotypes were identified in this study based on their MLVA profiles (Table A4). These genotypes were predominantly grouped according to the province and year of sampling. Notably, similarities were observed between the genotypes identified in this study and those found in neighboring countries across the Americas. However, the province of Cotopaxi was over-represented compared to other provinces, where fewer samples were collected, possibly contributing to its higher apparent diversity.

One genotype found in Pichincha (M1–M4) and Cotopaxi (M42 and G2) shared the same profile as genotypes reported by the United States Department of Agriculture (USDA) labeled as “SRR3096419” and first identified in 2016 (NCBI: https://microbesgenotyping.i2bc.paris-saclay.fr/databases/view/61/, accessed on 20 July 2024).

The isolates from samples collected in Tungurahua (M5, M6, and M7), Cotopaxi (M25, M26, M27, and M28), and Azuay (G3) matched bovine isolates from Brazil reported by [9] (NCBI: https://microbesgenotyping.i2bc.paris-saclay.fr/databases/view/61/, accessed on 20 July 2024). Within each province, the samples clustered into the same genotype, but each province was linked to a distinct strain of *B. abortus* biovar 1. Several studies have similarly identified *Brucella abortus* biovar 1 in Brazil [9,36,37]. According to the records of the Animal Health Certification Department of AGROCALIDAD, Ecuador has trade agreements involving the exchange of breeding cattle and embryos, which could have introduced *B. abortus* biovar 1 from Brazil into Ecuador [38]. The importation of infected animals from different regions or countries has been shown to introduce new genotypes into a particular region [39].

The presence of three distinct biovars of *B. abortus* in Ecuador is likely linked to the movement of livestock, which has facilitated the introduction and dissemination of *Brucella* strains across regions [24]. Given the genetic stability of *Brucella* species, it is more plausible that the observed diversity reflects introductions from external sources rather than recent local genetic mutations or recombination events [8]. Studies have shown that the MLVA assay primarily targets intergenic regions containing tandem repeats, which are not directly linked to specific genetic traits or coding regions [7]. Consequently, while MLVA provides valuable epidemiological insights, its results should not be interpreted as indicative of functional genetic adaptations. Addressing these historical and epidemiological factors is essential to understanding the diversity of *Brucella* genotypes in Ecuador. Strains of *B. abortus* biovar 1 from human cases in Ecuador showed certain peculiarities during typing, including growth inhibition on media colored with safranin (100 µg/mL) and fuchsin (20 µg/mL) [17], which may have hinted at the existence of different genotypes of *B. abortus* biovar. 1, as confirmed by the MLVA-16 results.

Other samples isolated from Cotopaxi province (M21, M22, M23, M24, M30, M31, M32, M33, and M50) were genotyped as *Brucella abortus* biovar 2, closely related to two isolates found in Brazil [9]. The continued importation of cattle from areas where this biovar is endemic may explain the first occurrence and isolation of *B. abortus* biovar 2 in this study [9,24,25]. This biovar was also reported in Argentina [33].

The two samples isolated in Cotopaxi province (M43 and G19), genotyped as *Brucella abortus* biovar 4, were similar to samples reported in Brazil in 2013 by Minharro et al. [9]. Although *B. abortus* biovar 4 is not the most common in Latin America, its presence has been documented in Brazil, Argentina [9], and Ecuador [17]. Monitoring this biovar requires broader brucellosis surveillance efforts across the continent.

The isolates collected in Santo Domingo (M36), Morona Santiago (G15), and Cotopaxi (G17) provinces share the same genotype as a circulating strain found in Costa Rica [26]. Hernández-Mora et al. (2017) reported that local strains of *B. abortus* found in Costa Rica are related to the circulating strains in North America and Brazil, with cattle in Costa Rica originating from these countries [26]. Given the similarities between the Ecuadorian and Costa Rican strains observed in this study, it is likely that a comparable relationship exists between circulating *B. abortus* biovar 2 strains in the Americas, including Ecuador [26].

Analyzing the frequencies of the loci reveals that certain loci, such as Bruce06 = 4, Bruce08 = 5, Bruce11 = 3 or 4, Bruce12 = 12, and Bruce43 = 8, are commonly found among the *B. abortus* strains in Ecuador. These loci may serve as indicators of specific genetic traits in the strains present in the country. The recurrence of these profiles across different provinces suggests that the strains are likely being spread through cattle movement or other transmission routes within Ecuador. In countries with a high prevalence of brucellosis, transporting infected animals between regions can facilitate the spread of the disease at both local and national levels. This issue is exacerbated in areas where the identification of infected animals is inefficient, or veterinary controls are inadequate [40]. The unregulated movement of infected animals facilitated the spread of *Brucella*, introducing the disease into previously unaffected populations and posing significant risks to animal and public health [24]. Understanding the dominant *Brucella* species and biovars affecting livestock is essential for developing effective strategies to prevent and control brucellosis in animal populations [41]. The detection of *B. abortus*, including strains associated with biovars 1, 2, and 4, in Ecuadorian livestock highlights the genetic diversity of the pathogen. Ecuador currently faces critical challenges regarding the quality of brucellosis vaccines and the effectiveness of field vaccination programs. The lack of rigorous quality control for vaccines has been identified as a major deficiency, and recent studies indicate that vaccines available in the country do not always meet the standards recommended by international organizations, potentially leading to inadequate immunization of animals (Garrido-Haro A., personal communication). Factors such as the variability of animal immunity, vaccine administration under field conditions, and general vaccination protocols likely play a more important role in vaccine efficacy than genetic differences between strains. This low vaccine efficacy allows virulent strains of *B. abortus* to persist within the bovine population, thereby increasing the incidence of the disease and complicating efforts toward its control. The absence of a mandatory and standardized vaccination program further exacerbates this challenge. To improve control efforts, it is important to implement a mandatory annual vaccination program for all young replacement females, ensure rigorous quality control of vaccines, and enhance training for veterinary professionals to guarantee proper vaccine handling, administration, and recording.

In Ecuador, brucellosis remains a significant public and veterinary health issue, largely due to the insufficient regulation of agricultural and livestock practices. Addressing these systemic gaps in vaccination quality, coverage, and disease management is vital for reducing the prevalence of brucellosis and improving animal and public health outcomes [16].

It is, therefore, necessary to implement adapted surveillance and control strategies across the country to achieve the effective detection, characterization, and control of circulating genotypes. Regions with more intensive surveillance tend to report greater genetic diversity, providing valuable insights into the specific measures within brucellosis control programs [27]. To address this, the government should allocate appropriate resources to the National Brucellosis Control Program, which could include mandatory vaccination, compensation for the slaughter of infected animals, quarantine regulations for newly introduced animals, and improved training and education for stakeholders. Furthermore, identifying and comparing local circulating genotypes of *Brucella* spp. with global strains can enhance the understanding of brucellosis epidemiology in Ecuador, facilitating the development of more effective control measures. The results of the present study can be shared with AGROCALIDAD, the bovine technical committee (*Mesa Consultiva Ténica Bovina*), veterinary professionals and technicians, veterinary supply stores, and farmers to raise awareness about the epidemiology of bovine brucellosis. This would help improve disease management practices across the country.

The impact of brucellosis in Ecuador highlights the urgent need for a more effective education–communication strategy and greater collaboration under the One Health approach, which integrates human health, animal health, and environmental sectors. While human cases of brucellosis have been recorded (likely under-reporting) in recent years (44 cases in 2019, 2 in 2020, 12 in 2022, 21 in 2023, and 27 in 2024), a more coordinated approach is still needed to prevent and control the disease [42].

One of the main challenges lies in the lack of a comprehensive educational strategy to raise awareness among both the population and workers in the agricultural sector about the risks of the disease and preventive measures. While the Ministry of Public Health is responsible for treating human cases, the active involvement of the Ministry of Agriculture and Livestock is crucial for prevention in the animal sector. The One Health approach aims to integrate these actions, promoting cooperation between the two ministries and other key institutions to reduce zoonotic transmission risks.

Greater collaboration, especially in rural areas and slaughterhouse zones where outbreaks are more common, is essential to improve surveillance and the prevention of brucellosis. Education and training in good agricultural and animal handling practices, along with public awareness about the safe consumption of animal products, could significantly reduce brucellosis cases in Ecuador.

## 5. Conclusions

The present study enabled the identification of Ecuadorian isolates of bovine *Brucella abortus* at the biovar level, using conventional and molecular techniques on samples collected from 2022 to 2024 and from six provinces in continental Ecuador.

The findings highlight the importance of continued surveillance and detailed genetic analysis to better understand the epidemiology of *Brucella* in different regions. Notably, the unexpected identification of *B. abortus* biovar 2 for the first time in Ecuador has significant public health and livestock implications. In this context, the results of this study suggest that the dynamics of herd movement between countries in the Americas may be an important factor in the spread of *Brucella* in the region. Although the study does not address herd movements, animal trade and movement practices are known to be key factors in the spread of bovine brucellosis, particularly in areas with inadequate biosecurity measures. In addition, previous studies have shown that mixing animals of different origin or age facilitates disease transmission and that cross-border, unregulated movements between countries may play a crucial role in the spread of the pathogen. Therefore, future studies should further investigate these aspects to identify herd movement patterns and assess their impact on the epidemiology of brucellosis in Latin America.

The complementary use of Farrell’s and CITA media maximized the efficiency, specificity, and reliability of *Brucella* isolation, which is crucial for rapid and accurate diagnosis of brucellosis. Identifying these isolates at the biovar level provided valuable genetic information that can serve as reference strains for future research. These findings offer a foundation for identifying undocumented biotypes in Ecuador and could be used in monitoring and follow-up programs for bovine brucellosis. Additionally, a new national sampling program should be considered to confirm the presence of biovars that may have been undetected or previously unidentified.

## Figures and Tables

**Table 1:** Summary of results of isolation and retromammary lymph node samples.

Sample Type	Province	Number of Samples	Medium Farrel	Medium Cita	PCR *Brucella abortus*
Milk	Pichincha	9	1	5	5
Milk	Tungurahua	3	3	3	3
Milk	Azuay	3	1	1	1
Milk	Cotopaxi	26	4	21	22
Milk	Santo Domingo	7	4	3	4
Retromammary lymph node	Morona Santiago	5	2	3	3
Retromammary lymph node	Azuay	5	2	3	4
Retromammary lymph node	Cotopaxi	10	3	6	8
Retromammary lymph node	Santo Domingo	4	4	3	4
Total	6	75	24	48	54

There were twice as many positive samples with the CITA medium (N = 48) compared to the Farrel medium (N = 24). The CITA medium appears to be more sensitive to a larger panel of main *Brucella abortus* that could be present in the samples.

**Table 2:** Summary of the MLVA-16 genotyping of *Brucella abortus* strains from Ecuadorian cattle.

Biovar/Group	Isolated	Genotypes	Herds
*B. abortus* biovar 1	9	4	3
*B. abortus* biovar 2	9	1	3
*B. abortus* group C-SRR309419	6	1	3
*B. abortus* C-Hernández-Mora 2017	3	2	3
*B. abortus* biovar 4	2	1	2

## Data Availability

The data that support the findings of this study are available from the corresponding author upon reasonable request.

## Appendix A

**Table A1:** Results of milk samples from cattle inoculated on Farrell and CITA culture media and identification of *Brucella abortus* through *Brucella abortus*-specific PCR and multiplex Bruce-ladder PCR.

Sample ID	Province	Year of Sampling	Farrell Medium	CITA Medium	PCR *Brucella abortus*	Bruce-Ladder
M1	Pichincha	2024	NI	+	+	*B. abortus*
M2	Pichincha	2024	NI	+	+	*B. abortus*
M3	Pichincha	2024	NI	+	+	*B. abortus*
M4	Pichincha	2024	NI	+	+	*B. abortus*
M5	Tungurahua	2024	+	+	+	*B. abortus*
M6	Tungurahua	2024	+	+	+	*B. abortus*
M7	Tungurahua	2024	+	+	+	*B. abortus*
M8	Azuay	2023	NI	NI	NI	NI
M9	Azuay	2023	NI	NI	NI	NI
M10	Azuay	2023	+	+	+	*B. abortus*
M11	Cotopaxi	2023	NI	NI	NI	NI
M12	Cotopaxi	2023	NI	NI	NI	NI
M13	Cotopaxi	2023	NI	NI	NI	NI
M14	Cotopaxi	2023	NI	NI	NI	NI
M15	Cotopaxi	2023	NI	+	+	*B. abortus*
M16	Cotopaxi	2023	+	+	+	*B. abortus*
M17	Cotopaxi	2023	NI	+	+	*B. abortus*
M18	Cotopaxi	2023	NI	+	+	*B. abortus*
M19	Cotopaxi	2023	NI	+	+	*B. abortus*
M20	Cotopaxi	2023	NI	+	+	*B. abortus*
M21	Cotopaxi	2023	NI	+	+	*B. abortus*
M22	Cotopaxi	2023	NI	+	+	*B. abortus*
M23	Cotopaxi	2023	NI	+	+	*B. abortus*
M24	Cotopaxi	2023	NI	+	+	*B. abortus*
M25	Cotopaxi	2023	NI	+	+	*B. abortus*
M26	Cotopaxi	2023	NI	+	+	*B. abortus*
M27	Cotopaxi	2023	NI	+	+	*B. abortus*
M28	Cotopaxi	2023	NI	+	+	*B. abortus*
M29	Cotopaxi	2023	+	+	+	*B. abortus*
M30	Cotopaxi	2023	NI	+	+	*B. abortus*
M31	Cotopaxi	2023	NI	+	+	*B. abortus*
M32	Cotopaxi	2023	NI	+	+	*B. abortus*
M33	Cotopaxi	2023	NI	+	+	*B. abortus*
M34	Santo Domingo	2022	+	+	+	*B. abortus*
M35	Santo Domingo	2022	NI	NI	NI	NI
M36	Santo Domingo	2022	+	NI	+	*B. abortus*
M37	Santo Domingo	2022	+	+	+	*B. abortus*
M38	Santo Domingo	2022	NI	NI	NI	NI
M39	Santo Domingo	2022	NI	NI	NI	NI
M40	Santo Domingo	2022	+	+	+	*B. abortus*
M41	Morona Santiago	2023	NI	NI	NI	NI
M42	Cotopaxi	2022	NI	+	+	*B. abortus*
M43	Cotopaxi	2023	+	NI	+	*B. abortus*
M44	Pichincha	2023	+	+	+	*B. abortus*
M45	Pichincha	2023	NI	NI	NI	NI
M46	Pichincha	2024	NI	NI	NI	NI
M47	Pichincha	2023	NI	NI	NI	NI
M48	Pichincha	2023	NI	NI	NI	NI
M49	Morona Santiago	2023	NI	NI	NI	NI
M50	Cotopaxi	2023	+	+	+	*B. abortus*

Abbreviations: ID, identification; NI, no isolation obtained; +, positive.

**Table A2:** Primers used in the Bruce-ladder multiplex PCR assay.

Primers	Sequence	Amplicon Size	DNA Target	Source of Genetic Differences
BMEI0998f	ATC CTA TTG CCC CGA TAA GG	1682	Glucosyltransferase, wboA gene	Insertion of IS711 in BMEI0998 in *B. abortus* RB51 and deletion of 15,079 bp in BMEI0993-BMEI1012 in *B. ovis*
BMEI0997r	GCT TCG CAT TTT CAC TGT AGC			
BMEI0535f	GCG CAT TCT TCG GTT ATG AA	450 (1320) ^a^	Immunodominant antigen, bp26 gene	Insertion of IS711 in BMEI0535-BMEI0536 in *Brucella* strains isolated from marine mammals
BMEI0536r	CGC AGG CGA AAA CAG CTA TAA			
BMEII0843f	TTT ACA CAG GCA ATC CAG CA	1071	Outer membrane protein, gene omp31	Deletion of 25,061 bp in BMEII826-BMEII0850 in *B. abortus*
BMEII0844r	GCG TCC AGT TGT TGT TGA TG			
BMEI1436f	ACG CAG ACG ACC TTC GGT AT	794	Polysaccharide deacetylase	Deletion of 976 bp in BMEI1435 in *B. canis*
BMEI1435r	TTT ATC CAT CGC CCT GTC AC			
BMEII0428f	GCC GCT ATT ATG TGG ACT GG	587	Erythritol catabolism, eryC gene	Deletion of 702 bp in BMEII0427-BMEII0428 in *B. abortus* S19
BMEII0428r	AAT GAC TTC ACG GTC GTT CG			
BR0953f	GGA ACA CTA CGC CAC CTT GT	272	ABC transporter-binding protein	Deletion of 2653 bp in BR0951-BR0955 in *B. melitensis* and *B. abortus*
BR0953r	GAT GGA GCA AAC GCT GAA G			
BMEI0752f	CAG GCA AAC CCT CAG AAG C	218	Ribosomal protein S12, rpsL gene	Point mutation in BMEI0752 in *B. melitensis* Rev.1
BMEI0752r	GAT GTG GTA ACG CAC ACC AA			

^a^ Includes the additional region introduced by IS711, which increases the total length of the amplicon.

**Table A3:** Results of retromammary lymph node samples from cattle inoculated on Farrell and CITA culture media and identification of *Brucella abortus* through *Brucella abortus*-specific PCR and multiplex Bruce-ladder PCR.

Sample ID	Sample	Province	Year of Sampling	Farrell Medium	CITA Medium	PCR *Brucella abortus*	Bruce-Ladder
G1	Retromammary node	Morona Santiago	2023	+	+	+	*B. abortus*
G2	Retromammary node	Azuay	2023	NI	+	+	*B. abortus*
G3	Retromammary node	Azuay	2023	+	+	+	*B. abortus*
G4	Retromammary node	Azuay	2023	+	NI	+	*B. abortus*
G5	Retromammary node	Cotopaxi	2023	+	+	+	*B. abortus*
G6	Retromammary node	Cotopaxi	2023	+	NI	+	*B. abortus*
G7	Retromammary node	Cotopaxi	2023	NI	+	+	*B. abortus*
G8	Retromammary node	Cotopaxi	2023	NI	NI	NI	-
G9	Retromammary node	Cotopaxi	2023	NI	+	+	*B. abortus*
G10	Retromammary node	Cotopaxi	2023	NI	NI	NI	-
G11	Retromammary node	Santo Domingo	2022	+	NI	+	*B. abortus*
G12	Retromammary node	Santo Domingo	2022	+	+	+	*B. abortus*
G13	Retromammary node	Santo Domingo	2022	+	+	+	*B. abortus*
G14	Retromammary node	Santo Domingo	2022	+	+	+	*B. abortus*
G15	Retromammary node	Morona Santiago	2023	+	+	+	*B. abortus*
G16	Retromammary node	Morona Santiago	2023	NI	NI	NI	-
G17	Retromammary node	Morona Santiago	2023	NI	+	+	*B. abortus*
G18	Retromammary node	Morona Santiago	2023	NI	NI	NI	-
G19	Retromammary node	Cotopaxi	2023	NI	+	+	*B. abortus*
G20	Retromammary node	Cotopaxi	2023	+	NI	+	*B. abortus*
G21	Retromammary node	Cotopaxi	2023	NI	+	+	*B. abortus*
G22	Retromammary node	Azuay	2023	NI	NI	NI	-
G23	Retromammary node	Azuay	2023	NI	+	+	*B. abortus*
G24	Retromammary node	Cotopaxi	2023	NI	+	+	*B abortus*
G25	Retromammary node	Pichincha	2024	NI	NI	NI	-

Abbreviations: ID, identification; NI, no isolation; +, positive.

**Table A4:** Results of the MLVA-16 genotyping of *Brucella* strains from Ecuadorian cattle.

ID Number	Province	Date of sample	Host	Bruce Number																Biovar of *B. abortus*
				06	08	11	12	42	43	45	55	18	19	21	04	07	09	16	30	
M1	Pichincha	2024	Cattle	4	5	3	12	2	2	3	3	6	43	8	3	6	3	5	6	*B. abortus*-groupC-SRR3096419
M2	Pichincha	2024	Cattle	4	5	3	12	2	2	3	3	6	43	8	3	6	3	5	6	*B. abortus*-groupC-SRR3096419
M3	Pichincha	2024	Cattle	4	5	3	12	2	2	3	3	6	43	8	3	6	3	5	6	*B. abortus*-groupC-SRR3096419
M4	Pichincha	2024	Cattle	4	5	3	12	2	2	3	3	6	43	8	3	6	3	5	6	*B. abortus*-groupC-SRR3096419
M5	Tungurahua	2024	Cattle	4	5	4	12	2	2	3	3	6	43	8	4	4	3	4	7	*B.abortus bv1*
M6	Tungurahua	2024	Cattle	4	5	4	12	2	2	3	3	6	43	8	4	4	3	4	7	*B.abortus bv1*
M7	Tungurahua	2024	Cattle	4	5	4	12	2	2	3	3	6	43	8	4	4	3	4	7	*B.abortus bv1*
M21	Cotopaxi	2023	Cattle	4	5	4	12	2	2	3	3	6	45	7	3	5	3	4	5	*B.abortus* bv2
M22	Cotopaxi	2023	Cattle	4	5	4	12	2	2	3	3	6	45	7	3	5	3	4	5	*B.abortus* bv2
M23	Cotopaxi	2023	Cattle	4	5	4	12	2	2	3	3	6	45	7	3	5	3	4	5	*B.abortus* bv2
M24	Cotopaxi	2023	Cattle	4	5	4	12	2	2	3	3	6	45	7	3	5	3	4	5	*B.abortus* bv2
M25	Cotopaxi	2023	Cattle	4	5	4	12	2	2	3	3	6	43	8	4	4	3	4	6	*B.abortus* bv1
M26	Cotopaxi	2023	Cattle	4	5	4	12	2	2	3	3	6	43	8	4	4	3	4	6	*B.abortus* bv1
M27	Cotopaxi	2023	Cattle	4	5	4	12	2	2	3	3	6	43	8	4	4	3	4	6	*B.abortus* bv1
M28	Cotopaxi	2023	Cattle	4	5	4	12	2	2	3	3	6	43	8	4	4	3	4	6	*B.abortus* bv1
M29	Cotopaxi	2023	Cattle	4	5	4	12	2	2	3	3	6	43	8	3	6	3	4	5	*B.abortus* bv1
M30	Cotopaxi	2023	Cattle	4	5	4	12	2	2	3	3	6	45	7	3	5	3	4	5	*B.abortus* bv2
M31	Cotopaxi	2023	Cattle	4	5	4	12	2	2	3	3	6	45	7	3	5	3	4	5	*B.abortus* bv2
M32	Cotopaxi	2023	Cattle	4	5	4	12	2	2	3	3	6	45	7	3	5	3	4	5	*B.abortus* bv2
M33	Cotopaxi	2023	Cattle	4	5	4	12	2	2	3	3	6	45	7	3	5	3	4	5	*B.abortus* bv2
M36	Santo Domingo	2022	Cattle	4	5	3	12	2	2	3	3	6	43	8	3	5	3	3	5	*B.abortus* group C (Hernández-Mora et al., 2017 [26])
M42	Cotopaxi	2022	Cattle	4	5	3	12	2	2	3	3	6	43	8	3	6	3	5	6	*B.abortus* group C-*SRR3096419*
M43	Cotopaxi	2023	Cattle	4	5	4	12	1	2	3	3	6	45	8	1	4	3	4	6	*B.abortus* bv4
M50	Cotopaxi	2023	Cattle	4	5	4	12	2	2	3	3	6	45	7	3	5	3	4	5	*B.abortus* bv2
G2	Azuay	2023	Cattle	4	5	3	12	2	2	3	3	6	43	8	3	6	3	5	6	*B.abortus* group C-SRR3096419
G3	Azuay	2023	Cattle	4	5	4	12	2	2	3	3	6	43	8	3	4	3	4	6	*B.abortus* bv1
G15	Morona Santiago	2023	Cattle	4	5	3	12	2	3	3	3	6	43	8	3	5	3	3	5	*B.abortus* group C (Hernández-Mora et al., 2017 [26])
G17	Morona Santiago	2023	Cattle	4	5	3	12	2	3	3	3	6	43	8	3	5	3	3	5	*B.abortus* group C (Hernández-Mora et al., 2017 [26])
G19	Cotopaxi	2023	Cattle	4	5	4	12	1	2	3	3	6	45	8	1	4	3	4	6	*B.abortus* bv4

Legend: The biovar of *B. abortus* was determined by comparing the results obtained for each locus with the public MLVA Bank *Brucella* v4_6_5 database (https://microbesgenotyping.i2bc.paris-saclay.fr/databases/view/61/, accessed on 20 July 2024). In cases where a biovar was not documented in the literature, the corresponding group from the MLVA Bank *Brucella* v4_6_5 was noted and cited with supporting references. Abbreviations: bv, biovar, SRR3096419: sample name (https://www.ncbi.nlm.nih.gov/sra/SRR3096419, accessed on 18 August 2024).
